# The role of the World Guidelines for Falls Prevention and Management’s risk stratification algorithm in predicting falls: a retrospective analysis of the Osteoarthritis Initiative

**DOI:** 10.1093/ageing/afae187

**Published:** 2024-08-22

**Authors:** Francesco Saverio Ragusa, Giovanna Di Bella, Ligia J Dominguez, Nicola Veronese, Lee Smith, Mario Barbagallo

**Affiliations:** Department of Health Promotion, Mother and Child Care, Internal Medicine and Medical Specialties “G. D'Alessandro”, Geriatric Unit, University Hospital Policlinic Paolo Giaccone, 90100 Palermo, Sicilia, Italy; Department of Health Promotion, Mother and Child Care, Internal Medicine and Medical Specialties “G. D'Alessandro”, Geriatric Unit, University Hospital Policlinic Paolo Giaccone, 90100 Palermo, Sicilia, Italy; Faculty of Medicine and Surgery, “Kore” University of Enna, 94100 Enna, Italy; Department of Health Promotion, Mother and Child Care, Internal Medicine and Medical Specialties “G. D'Alessandro”, Geriatric Unit, University Hospital Policlinic Paolo Giaccone, 90100 Palermo, Sicilia, Italy; Centre for Health Performance and Wellbeing, Anglia Ruskin University, Cambridge, UK; Department of Health Promotion, Mother and Child Care, Internal Medicine and Medical Specialties “G. D'Alessandro”, Geriatric Unit, University Hospital Policlinic Paolo Giaccone, 90100 Palermo, Sicilia, Italy

**Keywords:** fall, algorithm, prediction, osteoarthritis, older people

## Abstract

**Introduction:**

Recurrent falls are observed frequently among older people, and they are responsible for significant morbidity and mortality. The aim of the present study was to verify sensitivity, specificity and accuracy of World Guidelines for Falls Prevention and Management (WGFPM) falls risk stratification algorithm using data from the Osteoarthritis Initiative (OAI).

**Methods:**

Participants aged between 40 and 80 years were stratified as ‘low risk’, ‘intermediate risk’ or ‘high risk’ as per WGFPM stratification. Data from the OAI cohort study were used, a multi-centre, longitudinal, observational study focusing primarily on knee osteoarthritis. The assessment of the outcome was carried out at baseline and during the follow-up visit at 24 months. Data about sensitivity, specificity and accuracy were reported.

**Results:**

Totally, 4796 participants were initially included. Participants were aged a mean of 61.4 years (SD = 9.1) and were predominantly women (58.0%). The population was divided into three groups: low risk (*n* = 3266; 82%), intermediate risk (*n* = 25; 0.6%) and high risk (*n* = 690; 17.3%). WGFPM algorithm applied to OAI, excluding the intermediate-risk group, produced a sensitivity score of 33.7% and specificity of 89.9% for predicting one or more falls, with an accuracy of 72.4%.

**Conclusion:**

In our study, WGFPM risk assessment algorithm successfully distinguished older people at greater risk of falling using the opportunistic case finding method with a good specificity, but limited sensitivity, of WGFPM falls risk stratification algorithm.

## Key points

- We applied the World Guidelines for Falls Prevention and Management (WGFPM) algorithm to predict falls in the Osteoarthritis Initiative (OAI) study.
- The prevalence of reported falls at follow-up was significantly higher in those at ‘high risk’ than those at ‘low risk’.
- The algorithm produced a limited sensitivity, but a good specificity in predicting falls.

## Introduction

Falling is common among older people. Data reported that risk of suffering a fall progressively increases with age in the following manner: 60–69 years (34%), 70–79 years (49%), 80–89 years (65.5%) and above 90 years (63%) [1]. It has been widely assumed that osteoarthritis (OA) is an established risk factor for falls in older people [2, 3].

Predicting falls in those without a prior event is critical for primary prevention of injuries. Identifying and intervening before the first fall may be an effective strategy for reducing the high personal and economic costs of falls among older adults [4]. Although several falls risk assessment tools are available, it is unclear which have been validated and which would be most suitable for primary care practices. A systematic review [5] indicated that the most used falls risk assessment tools in clinical practice are Timed Up and Go (TUG) test [6], Gait Speed test [7], Berg Balance Scale [8], Performance Oriented Mobility Assessment [9], Functional Reach test [10] and history of falls. However, these tools are mono-dimensional and characterized by different sensitivity and specificity thresholds [5]. Recently, the 2022 World Guidelines for Falls Prevention and Management (WGFPM) proposed a new algorithm to systematically stratify the risk of falls, among older people. The algorithm stratifies community-dwelling older adults into ‘low risk’, ‘intermediate risk’ and ‘high risk’ of falling, in which different preventive strategies are indicated [11].

To the best of our knowledge, this algorithm was practically applied only in one large epidemiological study including 5882 Irish participants and the authors reported no differences between intermediate and high-risk groups exist in terms of predicting the risk of falls [11]. No other study is currently available on this topic. Importantly, the prediction of the risk of falls with standardized methods is a public health priority. Given this background, the aim of the present study is to verify sensitivity, specificity and accuracy of WGFPM falls risk stratification algorithm using data from the Osteoarthritis Initiative (OAI). In this population, the present paper aimed to analyse how the algorithm could identify older people having knee OA or at high risk for this condition at higher, intermediate and low risk of falls, and determine its utility in predicting the risk of falls.

## Materials and methods

### Data source and subjects

The OAI cohort study is a multicentre, longitudinal, observational study including participants with knee OA or at high risk of developing it [12]. Participants, aged between 40 and 80 years, were recruited across four clinical sites in the United States of America (Baltimore, MD; Pittsburgh, PA; Pawtucket, RI; and Columbus, OH) between February 2004 and May 2006. The OAI created a public archive of data, biological samples, and joint images collected over time from a very well clinically characterized population of individuals comprised of two subgroups: [1] had knee osteoarthritis (OA) with knee pain for a 30-day period in the past 12 months or [2] were at high risk of developing knee OA (e.g. overweight/obese [body mass index, BMI ≥25 kg/m^2^], family history of knee OA). The baseline assessments of this longitudinal cohort study consisted of an initial eligibility assessment by telephone, a screening clinic visit and an enrolment clinic visit. A follow-up visit and data collection was repeated after 24 months.

All participants provided written informed consent. The OAI study was given full ethical approval by the institutional review board of the OAI Coordinating Centre, at the University of California in San Francisco, CA, USA.

### Outcome: incidence of falls

A fall is traditionally defined as ‘an event which resulted in a person coming to rest inadvertently on the ground or floor or other lower level’ [13].

The assessment of the outcome was carried out at baseline and during the follow-up visit at 24 months. Participants reported the number of falls experienced in the preceding 12 months by answering questions about how they felt during the past 12 months.

### Exposure: algorithm to detect falls

We used a recently proposed algorithm that identifies different levels of risk of falling, i.e. low, intermediate and high [14]. Briefly, the WGFPM falls risk stratification algorithm has two points of entry i.e. opportunistic case finding and adults presenting to healthcare with a fall or fall-related injury. For this study, the OAI Wave 1 health assessment was treated as an opportunistic case finding. The questionnaire used is fully reported in Supplementary Table S1. Briefly, the algorithm has one sensitive question (i.e. history of falls during the previous year), while no information about fear of falls or feeling unsteady is available in the OAI. About the falls’ severity, in the OAI database, information about injuries, multiple falls and frailty was available, whilst data about syncope or inability to get up from the land were not available. Finally, to divide participants into low and intermediate risk of falls the evaluation of gait speed was used.

### Control variables

We considered several factors at the baseline evaluation, for descriptive purposes, that considered risk factors for falls, including: demographic characteristics (age, sex, ethnicity); education level (tertiary/higher level or not); smoking status (categorized as actual vs. never/previous); BMI; education level (categorized as tertiary or higher vs. other levels); yearly income (more or less than 50 000$); use of alcohol in the past; physical activity, measured through the Physical Activity Scale for the Elderly scale (PASE) [15]; individual’s functional mobility was assessed with Gait Speed over 4 m [16]; presence of self-reported depression was defined using the Centre for Epidemiologic Studies Depression Scale (CES-D), a brief self-report questionnaire to measure depressive symptoms severity in the general population was also implemented [14].

Presence of frailty was identified using the criteria proposed by the Study of Osteoporotic Fracture (SOF) index [17] in which this condition was defined as the presence of at least ≥ two out of three of the following criteria: (i) weight loss ≥5% taking place between baseline and the follow-up examinations (at the baseline examination a BMI of less than 20 kg/m^2^, a common cut-off for identifying underweight people in older people, was used, since no information regarding weight changes were recorded). Weight and height were measured at baseline and during follow-up examinations by a trained nurse, (ii) the inability to rise from a chair five times without arm support (hereafter referred to as inability to carry out chair stands) and (iii) poor energy based on the SF12 questionnaire response of ‘little at a time’ or ‘none at a time’ to the question ‘in the past 4 weeks, did you have a lot of energy?’

Comorbidities were assessed using self-reported information and the severity of comorbidities was assessed using the Charlson Comorbidity Index score [18]. Finally, number of medications was also recorded.

### Statistical analyses

The demographic and clinical characteristics of the patients were reported as mean and standard deviations for continuous variables or frequency and percentage for categorical variables. Between-group comparisons were performed using analysis of variance (ANOVA) for continuous variables and the Pearson Chi square test for categorical.

The sensitivity and specificity of the WGFPM falls risk stratification algorithm in predicting people with one or more falls between baseline and follow-up visit at 24 months were calculated using the following definitions:—True positives: participants categorized as ‘high risk’ who reported having fallen between baseline and follow-up visit.—True negatives: participants categorized as ‘low risk’ who reported no falls between baseline and follow-up visit.—False positives: participants categorized as ‘high risk’ who reported no falls between baseline and follow-up visit.—False negatives: participants categorized as ‘low risk’ who reported having fallen between baseline and follow-up visit.

Sensitivity and specificity were calculated either by ignoring the ‘intermediate risk’ group or by combining the intermediate- and high-risk groups.

A *P* < 0.05 was deemed statistically significant. All *posthoc* tests used Bonferroni adjustment for multiple comparisons. Analyses were performed using SPSS 26.0.

## Results

Among the 4796 participants initially included, we excluded 484 since no information about falls at the baseline were present and 331 since no information about falls at follow-up was available. Therefore, 3981 participants were included who were aged a mean of 61.4 years (SD = 9.1) and were predominantly female (58.0%). Using WGFPM falls risk stratification population was divided into three groups: low risk (*n* = 3266; 82%), intermediate risk (*n* = 25; 0.6%) and high risk (*n* = 690; 17.3%). Table 1 shows a description of the participants, by their risk of falls according to the WGFPM falls risk stratification. No significant differences were reported in relation to sex, age or ethnicity. As expected, patients at high risk of falls had higher BMI values (*P* < 0.001), higher CES-D scores (*P* < 0.001) and lower gait speed values (*P* < 0.001). These participants had often a higher prevalence of some diseases, such as asthma, or a higher comorbidities score and they usually used more medications than others (*P* < 0.001 for all comparisons). We also observed that patients at high risk of falls reported higher presence of injuries (*P* < 0.001).

**Table 1:** Sample characteristics by risk category.

Variable	Low risk (*n* = 3266)	Intermediate risk (*n* = 25)	High risk (*n* = 690)	*P*
General characteristics				
Female (*n*, %)	1871 (57.3)	17 (68)	420 (60.9)	0.72
Age (mean SD)	61.3 (9.0)	63.5 (9.9)	61.4 (9.4)	0.44
Whites (*n*, %)	2677 (82)	22 (88)	576 (83.6)	0.29
Falls last year (mean, SD) (*n*, %)	0.1 (0.3)	1 (0)	2 (0.7)	<0.001
Injury (*n*, %)	26 (0.8)	0	34 (4.9)	<0.001
BMI (mean, SD)	28.3 (4.7)	30.4 (4.6)	29.1 (4.9)	<0.001
Education to tertiary/higher level (*n*, %)	1028 (31.6)	5 (20)	232 (33.8)	0.56
Yearly income < 50 000$ (*n*, %)	2037 (67.9)	13 (52)	391 (59)	0.003
Actual smoke at baseline (*n*, %)	1476 (45.6)	14 (58.3)	331 (48.4)	0.15
Use of alcohol in the past (*n*, %)	1478 (45.5)	14 (56)	687 (49.9)	0.03
Evaluation tools				
Slow GS (*n*, %)	108 (3.3)	25 (100)	44 (6.4)	<0.001
PASE (mean, SD)	160.6 (80.1)	161.6 (78.2)	168 (87)	0.09
CES-D (mean, SD)	5.88 (6.2)	6.6 (5.4)	8.1 (8.1)	<0.001
Medical Conditions				
Frailty (*n*, %)	36 (1.1)	0	10 (1.5)	0.46
Heart attack (*n*, %)	52 (1.6)	1 (4.2)	21 (3.1)	0.007
Heart failure (*n*, %)	56 (1.7)	0	20 (3)	0.40
Peripheral artery bypass (*n*, %)	29 (0.9)	1 (4)	8 (1.2)	0.44
Stroke or TIA (*n*, %)	78 (2.4)	2 (8)	31 (4.6)	0.001
Asthma (*n*, %)	247 (7.7)	5 (20)	87 (13)	<0.001
Chronic lung disease (*n*, %)	60 (1.9)	1 (4.2)	22 (3.3)	0.016
Gastrointestinal ulcer (*n*, %)	80 (2.5)	0	24 (3.6)	0.12
Diabetes (*n*, %)	211 (6.6)	3 (12.5)	59 (8.8)	0.034
Cancer (*n*, %)	161 (4.9)	0	36 (5.2)	0.81
Arthritis (*n*, %)	1508 (46.6)	16 (64)	320 (46.6)	0.87
Comorbidity score (mean, SD)	0.3 (0.8)	0.3 (0.6)	0.5 (0.9)	<0.001
Medications (mean, SD)	3.4 (2.3)	5.3 (3.1)	4.2 (3)	<0.001

Abbreviations: BMI: Body Mass Index, GS: Gait speed, CES-D: Centre for Epidemiologic Studies Depression Scale, PASE: Physical Activity Scale for the Elderly, TIA: transient ischemic attack

At 24 months follow-up, the incidence of falls was as follows: among patients classified at low risk of fall, 820 experienced falls in the previous year (25.1%), 9 (36%) in the intermediate risk of falls group and 418 (60.6%) among those at high risk of falls, overall indicating a linear association between the different classes of risk and the real incidence of falls. As shown in Table 2, the WGFPM algorithm applied to OAI, excluding the intermediate-risk group, produced a sensitivity score of 33.7% and specificity of 89.9% for predicting one or more falls, with an accuracy of 72.4%. Combining participants in the intermediate- and high-risk groups, the WGFPM algorithm produced a sensitivity score of 34.2% and specificity of 89.5%, with an accuracy of 72.1%.

**Table 2:** Sensitivity and specificity analyses; ignoring or combining the intermediate-risk group.

	**True positives**	**True negatives**	**Sensitivity**	**Specificity**	**Accuracy**
**Intermediate-risk group removed from analysis**	418	2446	33.76%	89.99%	72.40%
**Combined intermediate-and high-risk group**	427	2446	34.24%	89.47%	72.17%

## Discussion

The aim of this study was to verify sensitivity, specificity and accuracy of WGFPM falls risk stratification algorithm using data from the OAI, analysing incidence of falls associated with WGFPM-derived intermediate- and high-risk groups, related to low risk. In our study, the WGFPM risk stratification algorithm efficaciously recognized people at higher risk of falling with the opportunistic case-finding process, but the real value of the intermediate-risk group stratification was not clear, likely owing to a small number of people in this group.

Falling status could be considered dynamic over time [19]. Different outcomes may represent a measure of ‘falls risk’: we analysed the clinical interpretation of this tool, any type of cut-off is quite arbitrary, and clinical significance of differentiating between dissimilar cut-offs (e.g. ‘1 or more falls’, ‘2 or more falls’ or ‘3 or more falls’) was uncertain, owing to people having a different perception of what a fall actually is. A previous study demonstrated how falls were usually under-reported when measured using a self-report recall tool, likely owing to a typical stigma connected to falls and dissimilarities in how falls are distinct and felt [20].

This algorithm indorses for the ‘intermediate risk’ group personalized exercises, teaching on falls risk and follow-up after one year. For those in the high-risk group evaluation of multifactorial risk, personal tailored interventions and revision in 30 to 90 days’ time [14]. As previously discussed, the ‘intermediate risk’ group was small and not significantly dissimilar to the high-risk group in terms of the number of falls.

This is a retrospective study, so we can only have partial interpretations why opportunistic case finding stratified only a small proportion of the OAI cohort as at intermediate risk. This could be the consequence of the Timed Up and Go (TUG) cut-off point >15 seconds that is identified as the same cut-off independently from some inherent characteristics, such as age or gender [21]. Generally speaking, a TUG time higher than 15 s, without presence of frailty, supposed syncope, or an injury caused by a fall in the last year, could be predictable to identify only a small part of the participants who fall in the future [22].

A decrease in gait speed is considered an early sign of worsening health and a useful method to detect vulnerable individuals early on, before any functional impairments become noticeable [23]. In our study, patients at high risk of falls had lower gait speed values, as previous showed by the literature. For example, in an American study of 2776 participants, the decline in gait speed over 12 months was associated with an increased likelihood of falls among community-dwelling older adults [24]. Similarly, in a Taiwanese study of 230 participants, fast gait speed was a protective factor for falls, even among subjects with history of falls, but slow gait speed was an independent risk factor even among subjects without history of falls [25]. Finally, in the same definition of frailty, a slow gait speed was recognized as an important and independent risk factor for falls [26].

Although its definition, the ‘low risk’ group still had a moderately high percentage of patients who reported falls: indeed, about a quarter of the participants included in this group reported a fall during the follow-up. In the OAI study, without considering the ‘intermediate risk’ group, the WGFPM algorithm had a low sensitivity with a high specificity and a good accuracy, overall meaning that ‘high risk’ in WGFPM risk stratification algorithm should be interpreted as expected to fall, whilst we cannot affirm that ‘low-risk’ group as not expected to fall. This finding, in our opinion, opens the question about if we can apply this algorithm as a screening tool in daily clinical practice to identify older people who prospectively fall. To the best of our knowledge, only a few studies reported data about this algorithm: differently from our study, the TILDA study included a general population, but found similar values to ours [11]; an American study of 1905 community-dwelling older adults, found that sensitivity and specificity were 45.3% and 83.4%, respectively [27], but there were some limitations, such as self-reported data, and potential confounders including frailty, that could be associated with fall risk, were not adjusted for. Moreover, there are limitations in relation to the population; indeed, participants were slightly younger, more likely to be white, married and live in a metro area. Therefore, our data demonstrated how the WGFPM algorithm had a good role avoiding false positive in a population affected by OA.

Finally, it should be noted that being considered as at high risk of fall by an algorithm may cause a fear of falling, could rise anxiety in older people and destabilize personality and independence [28]. At the same time, it may also cause changes in everyday life, like avoiding actions felt as high risk of falling [29]. Some people were incorrectly categorized as at low risk, and this may lead to a refuse of falls risk, avoiding their participation in falls prevention tools [30].

This study has some limitations. Firstly, the application of the WGFPM algorithm was feasible in our study, even if not completely (e.g. lying on the floor/unable to get up question was not available). Secondly, being at high risk of falls and falling are not the same things; identifying people in the high-risk group who did not fall as ‘false positives’ could be incorrect because these people may have lifestyle limitations and social alterations. Thirdly, falls were self-reported and this predisposed to bias due to recall and social fear of falls. Otherwise follow-up time of approximately 24 months is different from WFPM advices [14]; it is likely that an increased follow-up time intensified imprecisions in memories of falls. Fourthly, this is a retrospective study, so we can only have partial interpretations about why opportunistic case finding stratified only a small proportion of the OAI cohort as at intermediate risk. Finally, even if the OAI study includes people with knee OA or at high risk of this condition, the prevalence of some comorbidities increasing the incidence of falls is very low, which could affect the sensitivity and specificity found in our study.

In conclusion, WGFPM risk assessment algorithm successfully distinguished older people at greater risk of falling using the opportunistic case finding method. The usefulness of the intermediate-risk group was not clear and may not afford a real distinction with the high-risk group. This study showed a good specificity of WGFPM falls risk stratification algorithm using data from the OAI. Further prospective studies that include all the items included in the original version are necessary to better understand the feasibility and the accuracy of this algorithm in identifying older people at different risk of falls.

## Supplementary Material

aa-24-0474-File002_afae187
