# Supplementary material for: The role of the World Guidelines for Falls Prevention and Management’s risk stratification algorithm in predicting falls: a retrospective analysis of the Osteoarthritis Initiative
Source: Age Ageing. 2024 Aug 22;53(8):afae187. doi: 10.1093/ageing/afae187 (PMC11974246; doi:10.1093/ageing/afae187)
Supplement: aa-24-0474-File002_afae187 [file aa-24-0474-file002_afae187.docx]

**Supplementary Table 1. Application of the WGFPM risk stratification algorithm to the Osteoarthritis Initiative dataset.**

| **WGFPM risk stratification algorithm** | **Question** | **Variable used in the OAI dataset** |
| --- | --- | --- |
| **Assess falls in past 12 months (fall in last 12 months or positive answer to 3KQ)** | Has fallen in past year? | FU SAQ:Q17.Fallen and landed on floor or ground, past 12 months |
|  | Feels unsteady when standing or walking? | Not available |
|  | Worries about falling? | Not available |
| **Assess fall severity (answering ‘yes’ to one criteria is sufficient to satisfy severity criteria)** | Fall with injuries (severe enough to consult with a physician) | FU SAQ:*Q29a.Doctor said you broke or fractured lower arm or wrist bone(s), past 12 months ago  OR FU SAQ:*Q29a.Doctor said you broke or fractured lower leg or ankle bone(s), past 12 months ago |
|  | ≥2 falls last year | FU SAQ:Q17a.How many times fallen, past 12 months (calc) |
|  | Frailty | Combination between weight loss, poor energy, and slow chair time with > 2 indicating frailty |
|  | Lying on the floor/unable to get up | Not available |
|  | Loss of consciousness/suspected syncope | Not available |
| **Assess gait & balance** | Gait speed ≤0.8 m/s or Timed Up and Go (TUG) > 15 seconds | Gait speed ≤0.8 m/s |
